# Knowledge mapping of tumor microenvironment for breast cancer: a bibliometric analysis from 2014 to 2023

**DOI:** 10.3389/fimmu.2025.1550988

**Published:** 2025-03-27

**Authors:** Dongping Jiang, Ping Wen, Senmiao Zhang, Ningning Zhang, Qing Shao, Guanwen Wang, Long Wang, Sisi Li, Yang Qin, Fanli Qu, Xiaohua Zeng

**Affiliations:** ^1^ Chongqing University Cancer Hospital, School of Medicine, Chongqing University, Chongqing, China; ^2^ Department of Breast Cancer Center, Chongqing University Cancer Hospital, Chongqing, China; ^3^ Chongqing Key Laboratory for Intelligent Oncology in Breast Cancer (iCQBC), Chongqing University Cancer Hospital, Chongqing, China

**Keywords:** breast cancer, tumor microenvironment, bibliometric, immunotherapy, metastasis

## Abstract

**Introduction:**

Breast cancer is the most common malignant tumor among women worldwide, and the tumor microenvironment (TME) has become a hot research field in contemporary oncology. Understanding the TME is essential for elucidating the mechanisms of breast cancer progression and identifying novel therapeutic targets for metastasis and recurrence. This study performed a bibliometric analysis of TME research for breast cancer, providing a comprehensive overview of current research hotspots, development trends, and directions.

**Methods:**

We retrieved for literature on TME for breast cancer published during 2014-2023 from the Web of Science database and performed bibliometric analysis. CiteSpace was used for co-citation analysis of references to assess the evolution and bursts in the knowledge base. VOSviewer was used for co-occurrence analysis of author keywords, countries/regions, institutions, and authors to reveal the knowledge structure and collaborative networks in this research area. Thematic evolution trends were analyzed using the bibliometrix package to reveal research hotspots, thematic evolution and dynamic changes of this research.

**Results:**

A total of 9683 documents were included in this study, and the keyword co-occurrence analysis displayed five clusters: immunotherapy, metastasis, diagnosis and prognosis, metabolic regulation, and therapeutic approaches, with the first two keywords, immunotherapy and metastasis, being the most frequently mentioned. The most productive country/region, institution, and author were China (3266 publications), Shanghai Jiao Tong University (208 publications), and Takahashi, Kazuaki (37 publications).

**Conclusion:**

In breast cancer TME research, cancer-associated fibroblasts have attracted widespread attention, while cancer immunotherapy has emerged as a key focus in contemporary studies.

## 1 Introduction

Worldwide, breast cancer has become one of the most common malignant tumor in women. The International Agency for Research on Cancer published global cancer statistics for 2022, revealing that breast cancer, with 2.3 million new cases, was the second most common cancer worldwide, following lung cancer, and ranks fourth in mortality rate (1). Breast cancer is a heterogeneous disease with various molecular subtypes. Different molecular subtypes have distinct clinical characteristics and varying responses to treatment. For instance, HER2-positive and triple-negative breast cancer (TNBC) are highly prone to metastasis and have high recurrence rates. The treatment strategy for breast cancer has evolved from single surgical resection to a comprehensive therapy that includes chemotherapy, radiotherapy, targeted therapy, and immunotherapy. However, despite the continuous advancements in treatment methods for breast cancer, recurrence and metastasis remain the primary causes of treatment failure.

Lately, researchers have increasingly recognized that the development of breast cancer and the response to treatment are not only influenced by the intrinsic characteristics of tumor cells but are also closely related to the tumor microenvironment (TME) in which they reside. The TME is a complex micro-ecosystem composed of multiple immune cell types, cancer-associated fibroblasts (CAFs), endothelial cells, pericytes, and various other tissue-resident cells (2). Since Paget’s “seed and soil theory”, it has been recognized that tumor cell growth depends on its microenvironment (3). In 1993, Anderson and Whiteside formally introduced the concept of the “tumor microenvironment”, emphasizing the close relationship between tumors and their internal and external environments, with the TME capable of either promoting or inhibiting tumor development (4). Growing evidence suggests that the TME plays a critical role in the recurrence and metastasis of breast cancer. Immune cells, fibroblasts, and various signaling molecules within the microenvironment not only facilitate tumor cells in evading immune surveillance but also create conditions that promote their spread. Additionally, the complexity of the TME can contribute to resistance against conventional therapies, increasing the risk of treatment failure. Therefore, a deep understanding of the composition and function of the TME for breast cancer is essential for preventing recurrence and metastasis and improving treatment efficacy.

In recent years, a methodology known as the “research weaving” framework has been proposed, which uses bibliometrics and knowledge mapping techniques to visually present the hot spots and trends in a research field (5). Bibliometrics is a method that uses mathematical and statistical methods to quantitatively analyze knowledge carriers (usually literature) in a specific field. As a scientific research tool, bibliometrics has been widely applied in the development analysis of scientific research fields and has had a profound impact on the development of modern scientific research.

With the development of single-cell and spatial analysis technologies, the complexity and cellular heterogeneity of the TME have been gradually revealed, and its importance in tumor malignant progression, immune evasion, and therapeutic resistance has been increasingly being recognized. There is a growing number of academic publications in the field of TME for breast cancer, yet, faced with such a vast array of literature, there is a lack of bibliometric summaries and analyses of its research content and hotspots. In this study, publications related to TME for breast cancer were retrieved from the Web of Science (WoS) database, and the literature published from 2014 to 2023 was analyzed to present the research hotspots as well as the development direction of the field in a visual way, in order to facilitate subsequent researchers to quickly understand the field of TME for breast cancer and to provide guidance for the direction of future research.

## 2 Materials and methods

Searching strategy and data collection: On April 17, 2024, we searched for relevant literature between 2014 and 2023 in the field of TME for breast cancer using the Web of Science Core Collection (WoSCC). WoS is widely recognized as one of the most authoritative and reliable comprehensive academic literature database, as it includes taxonomic journals published by renowned publishers and institutions around the world (6, 7). The publications were limited to those written in English. The publication type was limited to articles or reviews. The search strategy used was TS = (“Breast Neoplasm*” OR “Breast Tumor*” OR “Breast Cancer*” OR “Cancer of the Breast” OR “Cancer of Breast” OR “Breast Carcinoma*”) AND TS = (“Tumor Microenvironment*” OR “Microenvironment*, Tumor” OR “Cancer Microenvironment*” OR “Microenvironment*, Cancer”); All relevant literature was retrieved and downloaded in “Plain text” format to avoid biases introduced by updates. Record content is fully recorded and quoted references.

We imported the retrieved data into CiteSpace (version 6.1.R6), VOSviewer (version 1.6.20), and the bibliometrix package (version 4.1.4) in R (4.4.0, https://www.r-project.org/) to perform bibliometric analysis.

CiteSpace (8), developed by Dr. Chaomei Chen based on Java language and citation analysis theory, is a citation visualization tool designed to analyze disciplinary development, research hotspots, and emerging trends. This study utilized CiteSpace to explored how research hotspots changed overtime by conducting a timeline view of co-cited reference, and then exported the top 20 references with the strong citation bursts. Metrics of significance were calculated by CiteSpace, including betweenness centrality, silhouette score, and modularity. Nodes with high betweenness centrality are represented with purple rings, indicating critical bridging roles (Q > 0.3 and S > 0.5 signify reliable clustering results). Analysis parameters were set for CiteSpace: Time slicing (2014–2023), years per slice (1), node type (cited reference), selection criteria (top N% = 10%), pruning (pathfinder and pruning sliced network), and all other settings remain default settings.

VOSviewer, developed by Van Eck and Waltman, is a bibliometric network construction and visualization tool widely used in literature knowledge mapping analysis (9). In this study, we use VOSviewer to build co-authorship and co-occurrence networks, including co-authorship analysis of countries/regions, institutions, and authors as well as co-occurrence analysis of author keywords, and calculated total link strength (TLS). TLS refers to the sum of the cumulative strength of all the links between two nodes in a network graph. The links between nodes can be cooperative relationship, co-occurrence relationship. In co-authorship network, TLS reflects the frequency or intensity of cooperation between two countries/regions, institutions, authors. In Co-occurrence analysis, TLS indicates the frequency that two keywords occur together. The size of nodes is proportional to the frequency of keywords, and different colors of nodes represent different clusters. In the visual map, the thickness and length between the nodes reflect connection strength and correlation.

Bibliometrix package is a powerful document network analysis tool based on R software, which developed by Cuccurullo C and Aria M (10). In this study, we use bibliometrix package to graph thematic evolution and thematic maps, which reflect the evolution in research directions and trends. Thematic evolution was displayed by Sankey diagrams to illustrate the evolution of a theme in different time slices, and thematic maps were a two-dimensional map which constructed with the density index as the ordinate and the centrality index as the abscissa. The thematic maps were divided into four quadrants according to the density value and centrality value. The first quadrant is Motor Themes, representing the core theme of high maturity. The second quadrant is Niche Themes, representing the themes highly developed but isolated themes. The third quadrant is Emerging or Declining Themes, representing a new or vanishing theme. The fourth quadrant is Basic and Transversal themes, which may become a research hotspot or a trend of future development. For the thematic evolution and thematic maps, the parameters used were weight index (inclusion index weighted by word-occurrences), time slices cutting year (2018), numbers of labels for each cluster (3). The software is also used to export relevant data, including Author’s H-index and G-index. Both indices can be used to assess the quantity and quality of academic output for researchers or countries/regions.

All the original data of this study were gained from January 01, 2014 to December 31, 2023 available in the public domain, and there was no involvement of human or animal studies or experiments. Therefore, ethical approval was not required.

## 3 Results

### 3.1 Annual growth trend of publications

**Figure 1:**
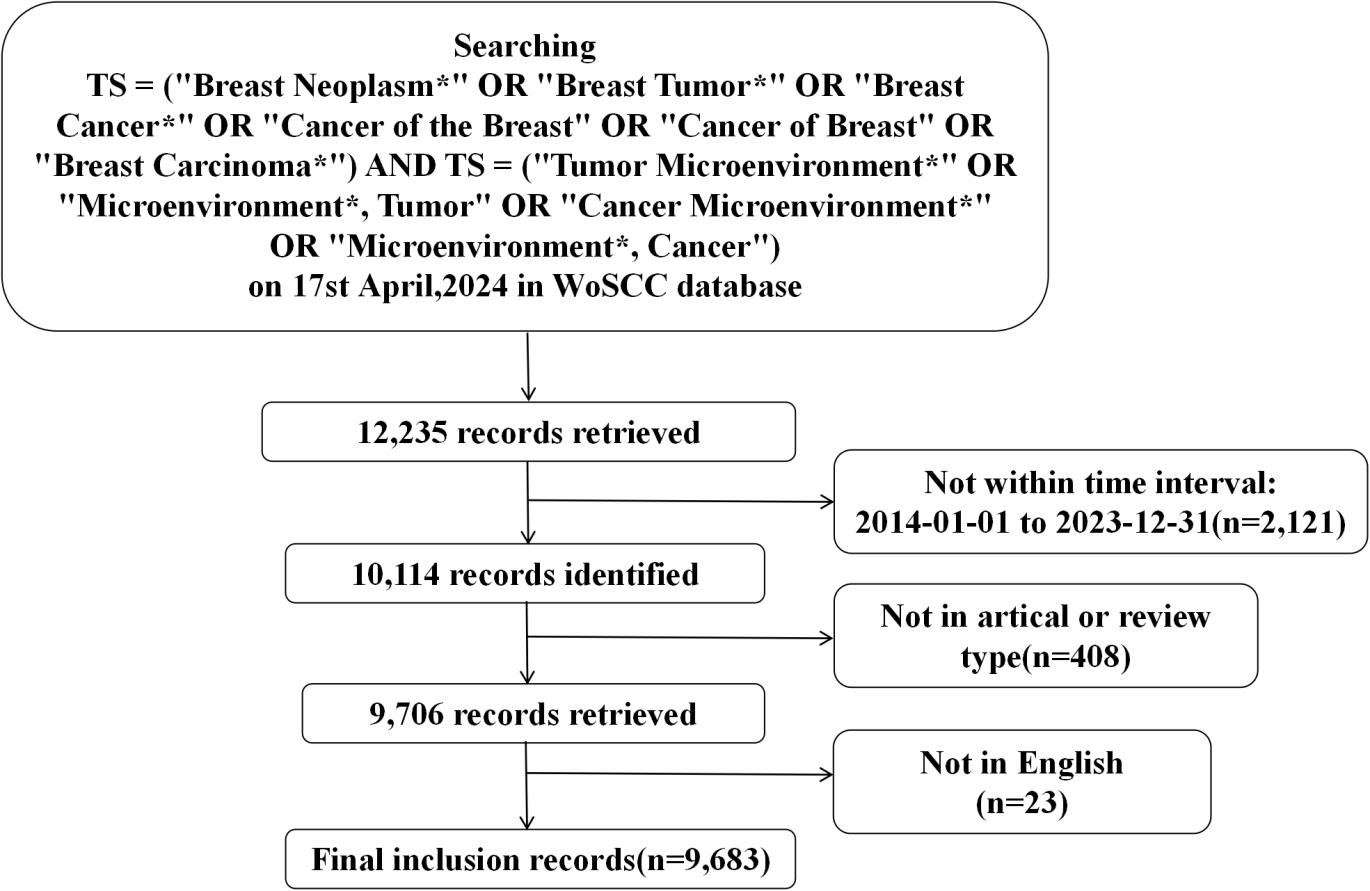
The flowchart of literature search and exclusion process.

**Figure 2:**
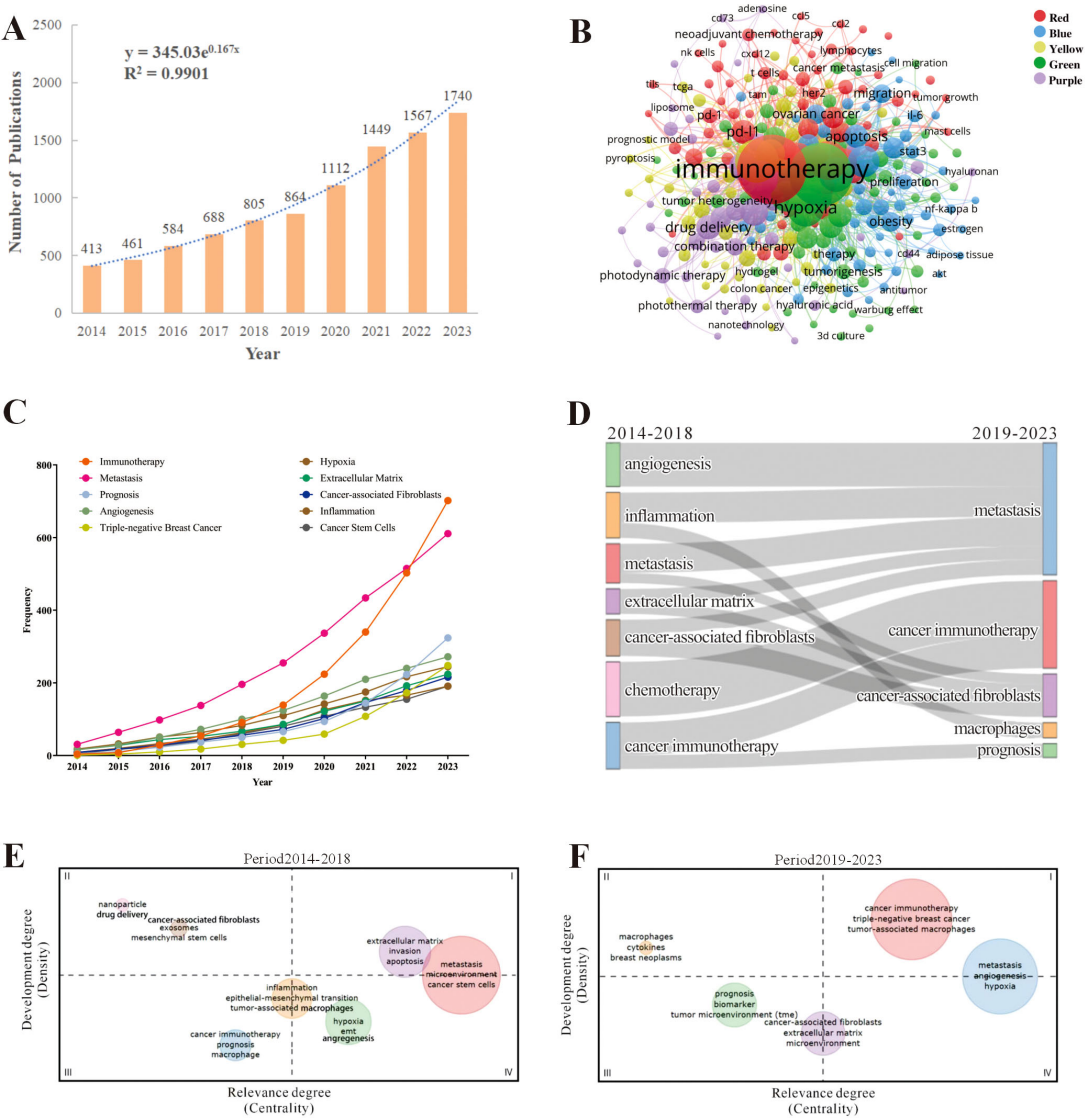
The hotspots and trends of publications in the field of tumor microenvironment for breast cancer. **(A)** The changes in the numbers of global publications over time; **(B)** The co-occurrence analysis of keywords, nodes are proportional in size to the frequency of keyword occurrence and the color of the nodes is determined by their category in cluster analysis; **(C)** The changes of high-frequency keywords over time; **(D)** The thematic evolution of publications in the last decade. Each node represents a research theme, and the connecting flows indicate how themes had transitioned over time. The width of each flow corresponds to the number of publications, with broader flows indicating a higher volume of research; **(E, F)** Thematic maps during 2014-2018 and 2019-2023. Thematic maps were divided into four quadrants: I: Motor Themes with high density and centrality II: Niche Themes with high density but low centrality III: Emerging or Declining Themes with low density and centrality IV: Basic and Transversal Themes with low density but high centrality.

A total of 9683 publications about TME for breast cancer that were published between 2014 and 2023 were obtained after searching the WoSCC database. Among these, 6590 (68%) were articles, and 3093 (31.9%) were reviews. The flowchart, as shown in **Figure 1** illustrated the literature search and selection process for excluded publications. During this period, the number of studies on the TME for breast cancer gradually increased by 321.3% from 413 publications in 2014 to 1740 publications in 2023 (**Figure 2A**). The total number of citations for all publications was 351262, with an average of 36.28 citations per publication. There was a significant increase in the number of publications in 2020 and 2021. We observed a statistically significant relationship between the year and the number of publications (R^2^ = 0.9901) according to the fitting data.

### 3.2 Analysis of keywords

To understand the research hotspots and topic distribution of TME for breast cancer, we employed VOSviewer to analyze the co-occurrence of the author’s keywords, as shown in **Figure 2B**. After removing the search term and synonyms, a total of 276 keywords were identified that had been used more than 18 times, and these keywords were formed five clusters. The red cluster, focused on TME and immunotherapy, indicates that research directions are focused on immune checkpoint inhibitors (ICIs), chemokines, tumor-infiltrating lymphocytes (TILs), and tumor-associated macrophages (TAMs). The green cluster centered on research related to metastasis within the TME for breast cancer, indicating that factors such as hypoxia, drug resistance, epithelial-mesenchymal transition, and CAFs can influence breast cancer metastasis. The yellow cluster concentrated on the diagnosis and prognostic assessment of breast cancer, where the prognosis of breast cancer can be analyzed through immune infiltration and biomarkers within the TME. The blue cluster illustrated the study of metabolic regulation in the TME for breast cancer, where lipid metabolism, glucose metabolism, and the associated signaling pathways are investigated. The purple cluster pertained to breast cancer therapeutic approaches related to the TME, including traditional chemotherapy, radiotherapy, immunotherapy, photodynamic therapy, photothermal therapy, nanotechnology, and drug delivery systems. We then exported the top 20 most frequent author keywords, as shown in **Table 1**. The main research keywords in this field include immunotherapy, metastasis, prognosis, angiogenesis, TNBC, hypoxia, extracellular matrix (ECM), CAFs, etc.

**Table 1:** Top 20 authors’ keywords of tumor microenvironment for breast cancer.

Rank	Keywords	Records	Total links	Rank	Keywords	Records	Total links
1	Immunotherapy	718	1715	11	Tumor-associated macrophages	187	501
2	Metastasis	611	1759	12	Drug resistance	182	504
3	Prognosis	327	783	13	Microenvironment	170	486
4	Angiogenesis	274	882	14	Biomarker	165	431
5	Triple-negative breast cancer	249	532	15	Cancer immunotherapy	162	305
6	Hypoxia	248	633	16	Exosomes	158	467
7	Extracellular matrix	224	696	17	Chemotherapy	154	418
8	Cancer-associated fibroblasts	218	589	18	Macrophages	144	419
9	Cancer stem cells	193	516	19	Cancer therapy	138	299
10	Inflammation	193	586	20	Drug delivery	125	252

### 3.3 Trends of themes

To understand the evolution of keyword-related topics, we analyzed the results of keyword frequency changes and their clustering. After removing synonyms, **Figure 2C** displays the dynamic trends of high-frequency keywords over time. We conducted a statistical analysis of the top 10 high-frequency keywords from the included publications, counting their annual occurrence frequencies. The keyword “Triple-negative breast cancer” saw its frequency rise from 1 occurrence in 2014 to 249 occurrences in 2023, indicating a 248% increase, making it the keyword with the highest growth rate. The term “immunotherapy” showed a consistently upward trend, sharply increasing from 91 occurrences in 2018 to 702 occurrences in 2023, becoming the most frequently used keyword and also ranking second in growth rate. The keyword “prognosis” gradually increased from 5 occurrences in 2014 to 324 occurrences in 2023, representing a growth rate of 63.8%. As shown in **Figure 2D**, the Sankey diagram illustrates the evolution of research themes over different time periods (2014-2018 and 2019-2023). Each node represents a research theme, and the connecting flows indicate how themes had transitioned over time. The width of each flow corresponds to the number of publications, with broader flows indicating a higher volume of research. During 2014-2018, there were seven thematic terms, which were angiogenesis, metastasis, ECM, CAFs, chemotherapy and cancer immunotherapy, while during 2019-2023, there were five key themes, the emerging theme words were macrophages and prognosis. From 2014-2018 to 2019-2023, inflammation was split into two clusters of metastases and associated with macrophages and breast cancer metastasis. In 2019-2023, studies revealed a close interaction between metastasis and angiogenesis, inflammation, ECM, and CAFs, indicating that these factors are closely related to the metastasis of breast cancer. Furthermore, CAFs not only interact with metastasis and the ECM but also have connections with cancer immunotherapy, which in turn is linked to chemotherapy. These findings suggest that, within the TME for breast cancer, CAFs promote metastasis through their interaction with the ECM.

The evolution of themes in different time periods based on centrality and density are shown in **Figures 2E, F**. In the comparison between the 2014-2018 and the 2019-2023, we noticed a substantial rise in both centrality and density of the “cancer immunotherapy” topic, signifying its importance and well-developed nature as it became part of the motor themes. Furthermore, from 2014-2018 to 2019-2023, the “angiogenesis” and “hypoxia” topics demonstrated a marked increase in their density and centrality. “Metastasis” has always been a hot topic in this field.

### 3.4 Evolution and burst of knowledge base

**Figure 3:**
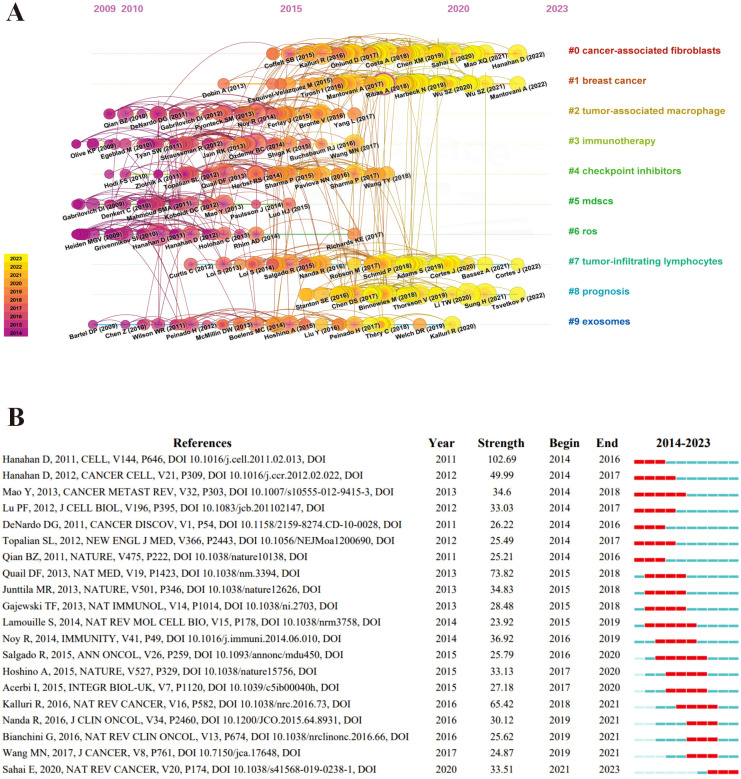
The burst of references co-citation in the field of tumor microenvironment for breast cancer. **(A)** The co-citation analysis of references in time-line manner. Nodes are proportional in size to the number of reference co-citations and the thickness and color of the node’s rings reflect the number of citations an article receives in a given year. Nodes with purple rings indicate high betweenness centrality, which are essential to connecting conceptual clusters that exist in different time periods. The connections between references are shown by the density of links and a unique color is assigned to each year. **(B)** The top 20 references with the strong citation bursts. A burst is a surge of the frequency of the citation of an article. The red bar indicates the time interval when the reference co-citation burst started and ended.

We performed a reference co-citation analysis to reflect the scientific knowledge repository in a timeline manner using CiteSpace, showing the largest 10 major clusters (**Figure 3A**). This picture exhibits every node symbolizing individual references; larger nodes denote a higher reference count. Nodes on the left represent earlier references, while those on the right represent more recent ones. Nodes positioned along the same line represent a cluster, identified by the label # on the right side. In the early stages, scholars focused their research on the field of TME for breast cancer on TAMs, immunotherapy, checkpoint inhibitions, myeloid-derived suppressor cells (MDSCs), ROS. In recent years, CAFs and prognosis have emerged as the main clusters. Exosomes and TILs are a long-standing cluster that has always been concerned by scholars.

According to the citation burst strength, we analyzed the top 20 references in the field of TME for breast cancer (**Figure 3B**). The publication with the most significant citation burst intensity was “Hallmarks of Cancer: The Next Generation”, a review led by Hanahan D’s group in 2011. This review reveals six biological abilities acquired during the development of human tumors, namely, sustaining proliferative signaling, evading growth suppressors, resisting cell death, enabling replicative immortality, inducing angiogenesis, and activating invasion and metastasis, providing a conceptual framework for understanding the tumorigenesis of breast cancer (11). The most recent of the top 20 cited references, published in 2020, primarily concerns the definition, origin, and role of CAFs in the TME (12), enhancing our understanding of fibroblasts within the TME for breast cancer.

### 3.5 Attribution and collaboration of countries/regions

**Figure 4:**
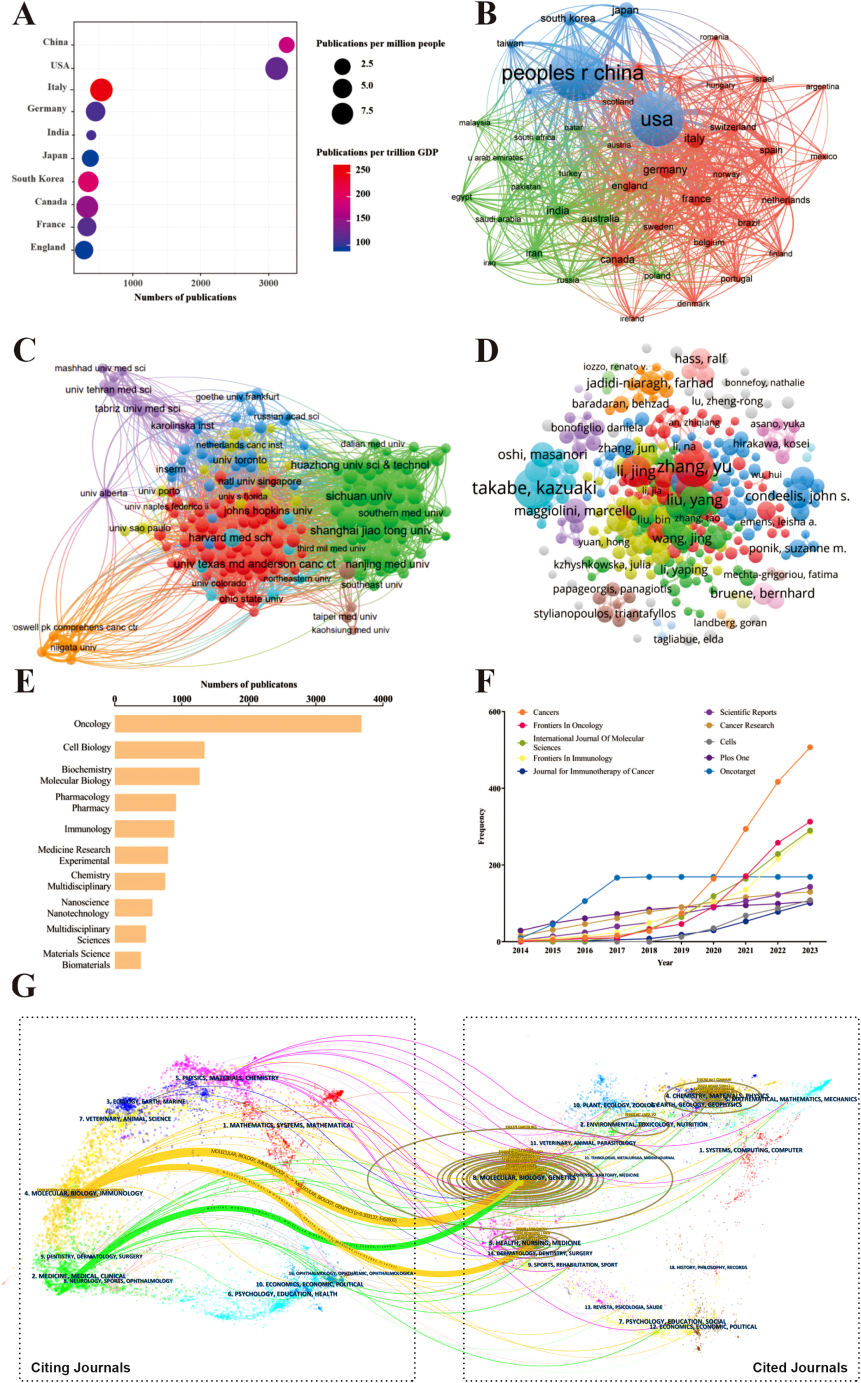
The attribution sources, collaboration networks in the field of tumor microenvironment for breast cancer. **(A)** The top 10 productive countries/regions in this field; **(B)** The co-authorship relationships of countries/regions, the size of the nodes indicates the number of publications, and the thickness and length of the links between the nodes indicate the strength and relevance of the connections between the nodes; **(C)** The co-authorship relationships of institutions; **(D)** The co-authorship relationships of authors; **(E)** The distribution of publications across disciplines; **(F)** The cumulative growth pattern of publications in the top 10 productive journals; **(G)** The dual-map overlay of journals, the left label in the figure represents citing journals, the right label represents cited journals, and the colored paths represent the citation relationships between them.

We used VOSviewer to analyze the countries and regions that have published papers in the field of TME for breast cancer. The analysis revealed that a total of 117 countries/regions have contributed to this field of which 45 countries/regions have published 25 or more papers in this area. Publication information classified by country or region was gathered and calculated (**Figure 4A**, **Table 2**). Among them, China had the highest number, totaling 3266 publications, which accounted for 33.73% of the total. It was followed by the United States with 3118 (32.20%). Italy had the highest number of publications per trillion gross domestic product (GDP), with a value of 266.38. The USA had the highest number of citations (152651) and the strongest link strength (1762). The collaboration relationships between countries/regions are described in **Figure 4B**. The countries/regions were divided into three colored clusters. Each cluster represented different collaboration patterns. The thickness of the links between nodes indicates the collaboration between countries/regions, calculated by the TLS. The largest blue cluster was centered on China and the United States and included several countries/regions, such as Japan, South Korea. The red cluster mainly consisted of European countries/regions, with Italy, Germany, England, France, Canada being the prominent centers. The green clusters mainly encompass India, Australia, Iran, Russia.

**Table 2:** The top 10 productive countries/regions in the field of tumor microenvironment for breast cancer.

Rank	Country/region	Numbers of publications	Publications per million People*	Publications per trillion GDP*	Numbers of citations	Average citations per publication	Co- authorship total link strength
1	USA	3118	9.36	122.56	152651	48.96	1762
2	PEOPLES R CHINA	3266	2.31	181.82	94337	28.88	804
3	GERMANY	449	5.36	109.98	18543	41.30	519
4	ENGLAND	280	4.18	90.64	14271	50.97	449
5	ITAIY	546	9.26	266.38	18604	34.07	431
6	CANADA	326	8.37	150.82	12531	38.44	370
7	FRANCE	322	4.74	115.87	17465	54.24	349
8	JAPAN	372	2.97	87.40	14616	39.29	243
9	INDIA	374	0.26	109.46	9812	26.24	240
10	SOUTH KOREA	333	6.45	198.93	10833	32.53	136

*Calculations based on 2022 population and GDP data from world bank (https://databank.worldbank.org/). GDP is calculated using GDP (current US$).

### 3.6 Attribution and collaboration of institutions and authors

The co-authorship relationships among these institutions, which had been grouped into ten clusters (**Figure 4C**). Details about the top 10 productive institutions were listed in **Table 3**. Shanghai jiao tong University held the highest number of publications (208) and the Harvard Medical School had the highest citation (13558 times) number and average citation per publication (98.96 times per publication). Chinese Academy of Sciences achieved the highest TLS (TLS = 256). Among all institutions, eight of the top 10 institutions are from China. These findings highlight China’s significant presence and contribution in this area of research.

**Table 3:** The top 10 productive institutions in the field of tumor microenvironment for breast cancer.

Rank	Institution	Country/ region	Numbers of publications	Numbers of citations	Average citation per publication	Co-authorship total link strength
1	Chinese Academy of Sciences	China	177	8627	48.74	256
2	Harvard Medical School	USA	137	13558	98.96	246
3	Shanghai jiao tong University	China	208	7015	33.73	173
4	The University of Texas MD AndersonCancer Center	USA	151	8968	59.39	163
5	Sun Yat-sen University	China	163	7994	49.04	154
6	Fudan University	China	170	5484	32.26	152
7	Nanjing Medical University	China	128	3966	30.98	110
8	Zhejiang University	China	139	3118	22.43	83
9	Sichuan University	China	185	6548	35.39	82
10	Huazhong University of Science and Technology	China	119	3080	25.88	71

The co-authorship relationships between scholars are illustrated in **Figure 4D**. A summary of the information regarding the top 10 most productive authors is presented in **Table 4**. Takabe, Kazuaki from Roswell Park Comprehensive Cancer Center have the largest number of publications (37), as well as the highest TLS score (128). Gao, Huile was identified as the author with the highest citation count (2066) and H-index (20), while Zhang, Yu had the highest G-index (36).

**Table 4:** The top 10 productive authors in the field of tumor microenvironment for breast cancer.

Rank	Author	Numbers of publications	Numbers of citations	Co-authorship total link strength	H-index	G-index
1	Takabe, Kazuaki	37	781	128	15	27
2	Oshi, Masanori	22	230	106	9	14
3	Condeelis, John S.	22	1671	63	15	23
4	Zhang, Yu	36	1849	43	19	36
5	Maggiolini, Marcello	22	709	42	15	22
6	Gao, Huile	22	2066	34	20	22
7	Liu, Yang	27	844	22	15	28
8	Li, Jing	28	465	16	13	21
9	Li, Wei	23	378	16	11	19
10	Zhang, Wei	26	585	14	12	24

### 3.7 Distribution across disciplines and journals

We performed a statistical analysis to recognize the top 10 subject categories which defined by the WoS classification in the publications (**Figure 4E**). The three most prominent disciplines within this field were Oncology, Cell Biology and Biochemistry Molecular Biology, accounting for approximately 65% of the total publications. The top 10 most prolific journals and the cumulative pattern of growth in annual publications are displayed in **Table 5** and **Figure 4F**, **respectively**. There were a total of 1342 academic journals publishing TME-related publications for breast cancer, with Cancers (n = 507, IF 2023 = 4.5) ranking first, followed by Frontiers in Oncology (n =313, IF 2023 = 3.5). The dual-map overlay of journals in **Figure 4G** demonstrated the topic distribution of the journals in this field. The journals that cite articles are located on the left side of the map, while the journals that are cited are on the right side. The labels represented the disciplines covered by the journals. Colored lines, moving from left to right, depict the citation pathways. In the figure, the vertical axis of the ellipse is proportional to the number of papers in the journal, and the horizontal axis of the ellipse is proportional to the number of authors. The subject of the source journal is represented by different link colors. There are three major citation pathways. Two orange citation paths suggest that journals from the Molecular/Biology/Genetics journals and Health/Nursing/Medicine journals were frequently cited in studies from the Molecular/Biological/Immunological journals. A green path demonstrates that studies from the Molecular/Biological/Genetic journals were frequently cited from studies in Medicine/Medical/Clinical journals.

**Table 5:** The top10 productive journals in the field of tumor microenvironment for breast cancer.

Rank	Source	Category	IF (2023)	JCR Category Quartile	Numbers of citations	Total publications	Average citation per publication	H-index	G-index
1	Cancers	Oncology	4.5	Q1	11208	507	22.11	50	83
2	Frontiers In Oncology	Oncology	3.5	Q2	7104	313	22.70	40	74
3	International Journal Of Molecular Sciences	Chemistry Multidisciplinary	4.9	Q1	8116	290	27.99	48	79
4	Frontiers In Immunology	Immunology	5.7	Q1	9945	286	34.77	55	91
5	Oncotarget	Cell Biology	5.168	Q2	7781	169	46.04	49	77
6	Scientific Reports	Multidisciplinary Sciences	3.8	Q1	4328	143	30.27	38	60
7	Cancer Research	Oncology	12.5	Q1	10859	130	83.53	57	102
8	Cells	Cell Biology	5.1	Q2	3286	108	30.43	29	54
9	Plos One	Multidisciplinary Sciences	2.9	Q1	3280	105	31.24	31	52
10	Journal For Immunotherapy Of Cancer	Oncology	10.3	Q1	3019	101	29.89	30	51

IF, impact factor; JCR, journal citation reports.

## 4 Discussion

The development of tumors is closely associated with their microenvironment, and breast cancer is no exception. In the microenvironment for breast cancer, various components interact and collectively influence tumor progression, metastasis, and treatment response. In this study, we primarily utilized the CiteSpace, VOSviewer, and bibliometrix packages to conduct a comprehensive overview of the field of TME for breast cancer. We retrieved 9683 studies published on this topic in academic journals from 117 countries/regions between 2014 and 2023, using the WoSCC database. Currently, research on the TME for breast cancer is on the rise. This study aims to explore the research hotspots, knowledge bursts, and thematic trends in the field from 2014 to 2023. We present our findings in a visual format to help researchers better understand the key focus areas and developmental trends in this field.

By analyzing the annual trends of keywords from 2014 to 2023, we observed significant growth in the keywords immunotherapy, prognosis, and TNBC, particularly from 2020 onward. This trend may be attributed to key developments in the field of the TME for breast cancer. Previous research considered breast cancer as “non-inflamed” or “cold”, characterized by low levels of TILs, PD-L1 positivity, and CD8+ T cells, which suggests a lower likelihood of response to immunotherapy (13). Subsequently, studies revealed that tumors in TNBC are relatively “hotter”, with more infiltrating lymphocytes (14), higher PD-L1 expression (15), and a greater tumor mutational burden, creating a favorable immune microenvironment for the application of ICIs (16). The clinical use of the PD-L1 inhibitor Atezolizumab (17) in 2019 and the PD-1 inhibitor Pembrolizumab (18) in 2020 has significantly improved the prognosis of TNBC patients and driven revolutionary advancements in breast cancer treatment.

In our keywords co-occurrence analysis, we identified a cluster related to breast cancer treatment methods involving the TME. These methods include traditional chemotherapy, radiation therapy, immunotherapy, photodynamic therapy. However, not all breast cancer patients benefit from these therapies, and identifying those who can benefit from treatment, with the help of components in the TME, is crucial. For all evaluated molecular subtypes, an increase in TILs density enhances responsiveness to neoadjuvant chemotherapy and correlates with prolonged survival in HER2-positive and TNBC patients (19). In addition, the abundance of TILs is closely associated with the prognosis of early-stage TNBC (20). TILs also predict trastuzumab efficacy in early-stage HER2-positive breast cancer (14). Moreover, elevated PD-L1 expression is associated with better immune therapy responses. For instance, in the IMpassion130 trial, PD-L1-positive metastatic TNBC patients treated with Atezolizumab and nab-paclitaxel demonstrated a more pronounced improvement in progression-free survival (PFS) (21). Similarly, in the KEYNOTE-355 trial, Pembrolizumab combined with various chemotherapy regimens improved PFS only in PD-L1-positive advanced TNBC patients (22).

Through the clustering timeline analysis of co-cited references, we observed that in the field of the TME for breast cancer, early clusters primarily focused on TAMs, immunotherapy, ICIs, MDSCs, and ROS. These foundational experimental studies have opened new avenues for breast cancer immunotherapy. TAMs and MDSCs are immunosuppressive myeloid cell components present in the TME. ROS not only mediates MDSCs-induced immunosuppression but also promotes the differentiation and polarization of the M2 phenotype through ERK and STAT3 activation (23, 24). Through the interpretation of the reference co-citation clustering timeline, it is evident that in recent years, CAFs and prognosis have emerged as major clusters of focus. Ana Costa et al. identified four CAFs subtypes with distinct characteristics and activation levels in breast cancer. It was found that CAF-S1 enrichment in TNBC may influence immunotherapy response and prognosis (25). Recent studies show that CAFs can undergo cellular senescence, forming senescent CAFs that promote tumor progression. These senescent CAFs contribute to the remodeling the microenvironment through the Senescence-Associated Secretory Phenotype, supporting local invasion and metastasis, and recruit innate immune cells that may suppress adaptive antitumor immunity (26). In the reference co-citation clustering timeline, we observed that TILs and exosomes have long been focal points in the study of the TME for breast cancer. Exosomes play a critical role in the TME as extracellular vesicles that mediate intercellular communication by transferring proteins, metabolites, and nucleic acids. They significantly alter the biological responses of recipient cells and can either promote or suppress disease progression. Exosomes derived from breast cancer cells promote metastasis by enhancing epithelial-mesenchymal transition via miR-200 (27) and integrins (28), and miR-122 facilitates metastasis by inhibiting pyruvate kinase and glucose uptake in lung cells (29). Exosomes are also involved in angiogenesis and ECM remodeling, key processes in tumor growth and metastasis. For example, miR-105 from breast cancer-derived exosomes disrupts endothelial tight junction protein ZO-1, compromising vascular integrity and increasing vascular permeability, thereby accelerating metastasis (30). Moreover, exosomes contribute to drug resistance mechanisms. HER2-positive exosomes, for instance, act as “decoys” for anti-HER2 therapies (31), limiting the efficacy of these treatments against cancer cells. As biomarkers and diagnostic tools, exosomes hold clinical potential for predicting metastasis and drug resistance. Future research should aim to elucidate the specific mechanisms of exosomes in breast cancer progression and explore their applications in diagnosis and therapy.

Through the analysis of thematic evolution, we found that angiogenesis, inflammation, ECM, and CAFs are closely linked to metastasis. Breast cancer-related mortality is primarily caused by distant metastases rather than the primary tumor (32). Angiogenesis plays a crucial promotive role in breast cancer metastasis, as tumor cells can utilize newly formed blood vessels to enter the bloodstream, thereby spreading throughout the body while receiving oxygen and nutrients to support their growth (33, 34). VEGFRs promote the formation of blood and lymphatic vessels by activating the JAK/STAT and PI3K/Akt pathways (35). Inhibiting VEGF signaling has been shown to suppress tumor growth in mice (36), but existing anti-angiogenic drugs still face challenges such as side effects and drug resistance, necessitating further optimization.

The analysis of thematic evolution reveals that inflammation within the TME can also influence breast cancer metastasis. Inflammation is also a significant factor in breast cancer metastasis. Research has found that IL-1β promotes lipid accumulation in lung mesenchymal cells (MCs), leading to metabolic reprogramming that impairs NK cell function and increases breast cancer metastasis to the lungs. Targeting lipid-rich MCs or blocking IL-1β may improve NK cell function and reduce breast cancer lung metastasis (37). Additionally, MCs synthesize prostaglandin E2 (PGE2) via prostaglandin-endoperoxide synthase 2 (PTGS2), resulting in neutrophil-mediated immunosuppression of T cells and NK cells. In mouse models, MC-specific PTGS2 deletion or PGE2 receptor inhibition alleviates immune suppression and significantly reduces breast cancer lung metastases (38).

The thematic evolution analysis clearly shows that CAFs and the ECM are also highly correlated with breast cancer metastasis. In stroma-rich breast cancers, CAFs remodel the ECM by secreting proteins such as collagen and fibronectin, establishing an immunosuppressive environment that facilitates tumor invasion and metastasis (39, 40). CAFs promote the degradation of normal ECM structure and the increase in matrix stiffness by secreting various matrix proteins and matrix metalloproteinases (MMPs) (41, 42), thereby facilitating tumor cell invasion and metastasis. In turn, ECM stiffening further enhances CAFs activation and function, creating a positive feedback loop (43). Studies have shown that YAP activation in CAFs regulates matrix stiffening, invasion, and angiogenesis (43),while FAK activation is linked to ECM stiffening and enhanced cancer cell invasion. FAK also correlates with increased immunosuppressive cells, reducing T cell activity (44). Thus, therapeutic strategies targeting CAFs, specifically by inhibiting the YAP and FAK signaling pathways, and limiting CAF-induced ECM remodeling, may prove to be an effective approach.

The thematic map reveals a significant increase in the centrality and density of the “cancer immunotherapy” theme, highlighting its prominence as a current research hotspot. Immune cells, cytokines, and other components of the microenvironment collectively influence its efficacy and tumor progression. Immunotherapy has shown great promise in treating breast cancer, particularly in TNBC. It leverages the patient’s immune system to target cancer cells and includes approaches such as ICIs, adoptive cell therapy, CAR-T therapy, cancer vaccines, cytokine therapy, tumor-infiltrating lymphocyte therapy, and oncolytic virus therapy, with ICIs being the most extensively studied. Components of the TME significantly influence immunotherapy. For example, dendritic cells within the TME present tumor-associated antigens and activate T-cell responses (45). Conversely, regulatory T cells (Tregs) in tumors suppress antitumor immune responses. Mouse models have shown that Tregs depletion alone is more effective in suppressing tumor growth than immune checkpoint blockade, and combining the two offers no additional benefit (46). These findings highlight the potential of modulating immune cells in the TME to improve breast cancer treatment. Additionally, the analysis of thematic evolution shows a considerable degree of cross-linkage between chemotherapy and immunotherapy. Both not only directly target tumor cells in breast cancer treatment but also play a crucial role by reshaping the TME. Chemotherapy can alter the number and function of immunosuppressive cells within the microenvironment, while immunotherapy enhances anti-tumor responses by activating or modulating immune cells. Together, they can work synergistically in breast cancer treatment. Chemotherapy modulates the immune microenvironment by depleting Tregs or enhancing the expression of major histocompatibility complex molecules, thereby improving T-cell function (47, 48). Studies have shown that combining chemotherapy with PD-1 inhibitors, such as pembrolizumab, significantly increases the pathological complete response (pCR) rate (49). For example, in the KEYNOTE-173 trial for TNBC, pembrolizumab combined with chemotherapy demonstrated a higher pCR rate with favorable safety (49). This suggests that targeting key factors in the TME through the combination of chemotherapy and immunotherapy holds great promise for more effective breast cancer treatment strategies.

China ranks first among the top ten most productive countries and regions, followed by the USA, which has the highest citation number and TLS, highlighting both countries’ significant contributions to TME research in breast cancer. Despite China’s higher publication count, its average citation per publication is lower, suggesting a need for greater academic innovation, stronger international collaborations, and enhanced academic impact.

This study has several limitations. First, only literature retrieved from WoSCC was analyzed, which may lead to incomplete data. Second, non-article and non-review publications were excluded, potentially introducing bias, though the number of such exclusions was relatively small, making the overall impact minimal. Lastly, the inherent limitations of bibliometric algorithms may overlook contributions from emerging researchers.

In conclusion, over the past decade, global research on the TME for breast cancer has increased rapidly. In the TME for breast cancer, CAFs, TAMs, TILs, and MDSCs have garnered significant attention, particularly CAFs, which have seen a rapid increase in interest over the past decade. Through in-depth research on the TME for breast cancer, it is hoped that the specific mechanisms underlying tumor progression, metastasis, and treatment resistance can be elucidated, thereby providing potential targets and theoretical foundations for personalized therapies and the development of novel treatment strategies for breast cancer.

## Data Availability

The original data supporting the results of this study are available from the corresponding authors upon request.
